# Effectiveness of low level laser therapy for treating male infertility

**DOI:** 10.1051/bmdcn/2018080207

**Published:** 2018-05-28

**Authors:** Sergey Vladimirovich Moskvin, Oleg Ivanovich Apolikhin

**Affiliations:** 1 O.K. Skobelkin State Scientific Center of Laser Medicine under the Federal Medical Biological Agency Moscow 121165 Russia; 2 N.A. Lopatkin Research Institute of Urology and Interventional Radiology Moscow 105425 Russia

**Keywords:** Male infertility, Sperm motility, Low level laser therapy

## Abstract

In half of the cases, the infertility of the couple is due to the disorder of the male fertility. The leading factors that cause male infertility are urogenital infections, disorders of the immune system, testicular and prostate pathology, as well as endocrine disorders.

Low level laser therapy (LLLT) is a very effective physical therapy method, used in many areas of medicine, including obstetrics and gynaecology, andrology and urology; and it is recommended as an integral part of the complex treatment of infertility.

The literature review showed that LLLT is beneficial in treating male infertility. Laser can significantly improve the survival, motility and speed of movement of spermatozoa. Laser therapy of patients with prostatitis and vesiculitis can eliminate infiltrative-exudative changes, improve reproductive and copulatory functions. Local illumination of red (635 nm) and infrared (904 nm) spectra should be combined with intravenous laser blood illumination (ILBI) of red (635 nm) and ultraviolet (UV) (365 nm) spectra.

## 1. Introduction

Translational medicine promotes a faster implementation of scientific achievements in the field of practical public health, allowing a personalization of treatment, which positively affects its results. This interaction was described as "Bench-to-Bedside" or "Bedside-to-Bench" [1]. This is an interdisciplinary field of modern medicine, based on the achievements of science: physiology, molecular biology, genetics and clinical research, created to ensure a higher efficiency of medical services.

Laser therapy is a vivid example of interdisciplinary medicine, which was based on the fundamental research in the field of physiology, biophysics and biochemistry, resulting in the emergence of highly effective therapeutic techniques that take into account the individual characteristics of the patient. However, it is only possible to see the full potential of laser therapy by strictly following the rules, approved by LLLT standards [2, 3] and using appropriate equipment.

Male infertility is a multifactorial syndrome that includes a wide range of disorders, a symptom of many different pathological conditions affecting both the sexual and other body systems: endocrine, nervous, blood, and immune [4-6].

According to the recommendations of World Health Organisation (WHO) (2000) [7], 16 main nosologies are distinguished, each of which, in turn, includes upwards of several dozen specific pathogenetic factors, 4 of 16 diagnoses are descriptive, without indicating the true cause: idiopathic oligo-, astheno-, terato- and azoospermia.

Sexually active couples, not protected during the year and not having had any children, according to WHO, are regarded as infertile. During the first year, about 25% of couples do not get pregnant. Of these, 15% seek medical help, and less than 5% do not succeed. In half of the cases, the infertility of the couple is due to the disorder of the male fertility. Causes of male infertility can be congenital or due to acquired abnormalities of the genitals, infections of the genitourinary system, increased scrotal temperature (varicocele), endocrine disorders, genetic abnormalities and immunological factors [8].

It is suggested that most idiopathic forms are genetically due to mutations and polymorphisms of many genes [4]. However, this hypothesis does not have rigorous proof and requires detailed studies [9]. Certainly, some pathologies are associated with a mutation, that is, damage to the DNA, but there is no doubt that in the overwhelming majority of cases, this is only the result of epigenetic changes in the genome that are reversible [10]. At the same time, it is known that low-intensity laser light not only effectively protects cells from DNA damage by various physical and chemical pathogenic factors, but is also able to activate "necessary" genes, which is often used in breeding [10]. This review by Miktadova A.V. *et al.* has dozens of references to prove this.

Data on the main causes of male infertility are extremely controversial [4-6, 12-15]. It is most likely that such a significant spread of data is due to differences in the methods of assessing the patients' condition, the diagnostic methods used, the presence or absence of various devices. Of course, the influence is also exerted by the country in which the research was conducted.

## 2. Causes of male infertility

However, we can confidently say that the leading factors that have the greatest impact on male fertility are the consequences of urogenital infections, including viral infections [16-18], and related disorders of the immune system, testicular and prostate pathology (varicocele, epididymoorchitis, prostatitis) [19-23], as well as endocrine disorders [24, 25]. Chronic nonspecific prostatitis, according to different data, causes infertility in 52-76% of cases [26-29].

Diagnosis of male infertility is based on a comprehensive assessment of the condition of the male reproductive system, conducted in a well-known sequence with the application of anamnestic, clinical, laboratory and special survey methods. To date, there is a variety of diagnostic methods of examination [8], although in general, diagnostic issues need a deeper and more comprehensive research.

The most important component of the treatment is the elimination of potentially harmful environmental factors, work and lifestyle. With some anomalies, for example, cryptorchidism, injuries, infections, the effects of toxic substances and medicines, infertility can be prevented. To restore the fertility of men, it is necessary:

- to establish a normal work and sleep cycle, receive adequate nutrition, treatment of concomitant diseases, adjust the rhythm of sexual life;
- to eliminate overheating, to reduce physical loads when practicing extreme sports;
- to eliminate the factors that cause depression, the state of fear and neuroses.

The fulfilment of these conditions in many cases may contribute to the improvement of the spermogram indexes [4, 5, 8], therefore, in most cases, the causes of the disease are nonspecific violations of physiological processes affecting spermatogenesis.

Unfortunately, etiotropic and pathogenetic treatments that give good results are, in most cases, inapplicable because it is impossible to unambiguously establish the specific cause of the disease and having a lack of knowledge regarding the mechanisms of the development of the disease. Bozhedomov V.A. *et al.* (2013) [4], criticize the "empirical", *i.e.,* nonspecific therapy for inefficiency (although not mentioning physical therapy, including low level laser therapy, as well as balneology), indicate the need for "tertiary prevention" with the aim of reducing complications after the application of other methods of treatment.

Attention is drawn to the fact that practically in any review of scientific literature and monographs on male infertility, there is no mention of physical therapy methods of treatment. Nevertheless, low level laser therapy, which has been actively developing in recent years, not only has almost no contraindications and side effects, but also has pronounced protective properties [11], and most importantly, demonstrates the highest effectiveness of treatment in many areas of medicine, including in obstetrics and gynaecology [30], andrology and urology [31-33] and is recommended as an integral part of the complex solution of the infertility problem [20], *i.e.,* it is successfully applied by those specialists who somehow face the problem of infertility.

In many cases, childless partnerships are a problem for couples [20], but it is quite obvious that in order to study the issues of interaction between the parties, it is necessary to understand as closely as possible the corresponding violations inherent in each sex, and also to justify the possibility of using low level laser therapy. Therefore, in this article, only male infertility is considered, but with the prospect of also studying the possible influence of low-intensity laser illumination (LILI) on female fertility, including within the framework of solving some issues arising during *in vitro* fertilization (IVF).

*Understanding the biomodulating processes that result from the absorption of LILI and the underlying methodology of low level laser therapy (LLLT), have allowed us to substantiate many methods, and also to optimize the already known ones in different fields of medicine.* The primary mechanism of the LILI biomodulatory action is the response of the organism to non-specific, that is, not associated with specific acceptors, absorption of laser light in various cells, resulting in a short-term increase in the concentration of calcium ions in the cytosol, the propagation of waves of increased Ca^2+^ concentration both in cells and in various biotissues. Following this, an organism response develops (secondary mechanisms), which begins with the activation of Ca^2+^ -dependent processes [2, 34].

## 3. Experimental research

The action of the laser beam for the study of the various physiological processes that determine, in particular, the motility of spermatozoa began almost from the moment of the appearance of lasers [35]. Numerous studies confirm the positive influence of LILI on the spermatozoa of various animals, their motility and the content of adenosine triphosphate (ATP) increases [36–59], cell life expectancy increases [60] as well as the probability of fertilization increases [61, 62]. But it must be noted that the research conditions were significantly different, and the parameters of the laser illumination technique are not accurately described (Table 1), which does not allow assessing the reliability and reproducibility of these results.

**Table 1:** Experimental studies on the effect of LILI on spermatogenesis and sperm quality.

**No**	**Experimental model**	**Results**	Illumination parameters*	**Reference**
1.	Stallions	Activation of the sexual reflexes of stallions (reduction of the time of preparation for the mount by 2-3 times and the number of mounts expended for 1 ejaculate), an increase in the activity of spermatozoa and rate of fertilization of mares.	1. 890 2. pulsed 3. 5-7 W 4. - 5. 500 6. 0, 5-1 (-) 7. - 8. 10 9. Daily 10. Laser acupuncture and externally on the testes	Adamkovskaya M.V., 2004 [36]
2.	Sperm, buffalo	Increase of the semen quality parameters. Maximum improvement was observed after 4 minutes of exposure	1. 532 2. continuous 3. 1 mW 4. 1,32 mW/cm^2^ 5. - 6. 1-5 7. 0.076, 0.15,0.23, 0.31, 0.38 J/cm^2^ 8. - 9. - 10. from above, homogeneously	Abdel-Salam Z. *et al,* 2011 [39]
3.	Sperm, dogs	Improvement and maintenance of sperm motility over time, decrease of the L-lactate production rate.	1. 655 2. - 3. 21,7 mW 4. - 5. - 6. 103/154/258 s 7. 4/6/10 J/cm^2^ 8. - 9. - 10. from above, homogeneously	Corral-Baqués M.I. *et al,* 2005 [40]
4.	Sperm, dogs	The most changes were observed when output power 49.7 mW was used: increase of the progressive velocity (VSL), average path velocity (VAP), linear coefficient (LIN), straightness (STR), wobble (WOB) and beat cross frequency (BCF) and reduction of the mean amplitude of lateral head displacement (ALH). The output power 6,84 mW was the only one to keep the same motility parameters 45 min after illumination.	1. 655 2. continuous 3. 6,8/15,4/33,1/49,7 mW 4. - 5. - 6. 7. 3,34 J (5.97 J/cm^2^) 8. - 9. - 10. -	Corral-Baqués M.I. *et al,* 2009 [41]
5.	Sperm, bulls	No differences in motility parameters. Increase in ROS generation (with 5 mW compared to 7.5 and 10 mW, and with 10 min compared to 5 and 1 min of illumination). Illumination with 5 mW caused more acrossomal/plasma membrane damage, and an increase in the number of cell with intermediate and higher mitochondrial potential.	1. 633 2. - 3. 5 / 7,5 / 10 mW 4. - 5. - 6. 1, 5, 10 7. 30, 150, 300 / 45, 230, 450 / 60, 300, 600 mJ/cm^2^ 8. - 9. - 10. through a spatial filter, omogeneously	Dreyer T.R. *et al.,* 2011 [43]
6.	Sperm, sea urchin	Effect on the locomotor activity of sperm, 2–5 times increasing the percentage of active cells depending on the time after exposure.	1. LED 650 / laser 635 2. - 3. - 4. 90, 250, 750 / 290 mW/cm^2^ 5. - 6. - 7. 0,07, 0,7, 7, 70 / 3 mJ/cm^2^ 8. - 9. - 10. 19 LEDs matrix at a distance of 10 cm / at a distance of 12,5 cm	Drozdov A.L. *et al,* 2014 [44]
7.	Sperm, Echiuroid, *Urechis unicinctus*	Enhancement of the respiratory rate of sperm in the presence of CO in proportion to the fluence rate. A sharp and large peak was observed at 430 nm, broad and small peaks at 530 and 570 nm.	1. 350 nm (fluence rate 7.7 x 1015 fotons/cm^2^/s) 400 (6.5 × 1015) 430 (9.9 × 1015) 450 (1.1 × 1016) 500 (1.0 × 1016) 530 (1.1 × 1016) 570 (1.0 × 1016) 600 (9.7 × 1015) 650 (7.9 × 1015)	Fujiwara A. *et al.,* 1991 [45]
8.	Sperm, rabbit	Illuminated samples during *in vitro* liquid storage better maintained motility, acrosome integrity and viability. Stimulation of the sperm mitochondrial respiratory chain increases the viability of sperm cells.	1. 633 2. continuous 3. 6 mW 4. - 5. - 6. - 7. 3.96, 6.12, 9 J/cm^2^ 8. - 9. - 10. -	Iaffaldano N. *et al,* 2010 [46]
9.	Sperm, frozen/thawed, chicken, pheasant and turkey	The possibility for restoration of motility of cryopreserved spermatozoa. Increase in sperm motility of turkey sperm, increase in COX activity in pheasant and turkey sperm	1. 633 2. continuous 3. 6 mW 4. - 5. - 6. - 7. 3,96, J/cm^2^ 8. - 9. - 10. -	Iaffaldano N. *et al,* 2013 [47]
10.	Sperm, bulls	Acceleration of Ca^2+^ transport. Laser can stimulate Ca^2+^ exchange through the cell membrane that causes transient changes in the cytoplasmic Ca^2+^ concentration which control motility and acrosome reaction in spermatozoa and can trigger mitosis in other cells.	1. 630 / 780 2. - 3. 35, 10 / 13, 40 mW 4. - 5. - 6. - 7. 2-30 J/cm^2^ 6. - 6. - 8. - 9. - 10. -	Lubart R. *et al.,* 1992 [48]
11.	Sperm, bovine	An accelerated Ca^2+^ uptake by the mitochondria after He-Ne and inhibition after high intensity illumination. The ATP-dependent Ca^2+^ uptake by the sperm plasma membrane vesicles was not changed by 633nm and was enhanced by 780 nm.	1. 633/780 2. - 3. 13/24 mW 4. 0,03-1,2 W/cm^2^ 5. - 6. 1-15 min 7. - 8. - 9. - 10. -	Lubart R. *et al.,* 1996 [49]
12.	Sperm, bulls	Inhibition of Ca^2+^ uptake by sperm mitochondria and enhancement of Ca^2+^ binding to sperm plasma membranes.	1. 780 ± 5 2. - 3. 3-25 mW 4. - 5. - 6. 1-20 min 7. - 8. 9. - 10. -	Lubart R. *et al,* 1997 [50]
13.	Human sperm, patients with Asthenzoo-spermia	Increase of progressive motility with 4 and 6 J/cm^2^ at the times of 60 and 45 min, respectively.	1. 830 2. continuous 3. 100 mW 4. - 5. - 6. 26,8 / 40.2 / 67 s 7. 4 / 6/ 10 J/cm^2^ 8. 9. -10. from above, homogeneously	Salman Yazdi R. *et al,* 2014 [54]
14.	Human sperm, normal subjects and patients with infertility disorders.	Stimulation of sperm motility (most effective with 32 J/cm^2^) but not velocity. Non-motile live spermatozoa probably are stimulated.	1. 647 2. - 3. - 4. - 5. - 6. 80 / 80/ 80/ 80/ 80/ 160 s 7. 0,5 / 1,0 / 2,0 / 4,0 / 8,0/ 32 J/cm^2^ 8. - 9. - 10. -	Sato H. *et al.,* 1984 [56]
15.	Sperm, bulls	Modulation of bovine sperm function by 10 min illumination, increase of motility parameters and mitochondrial potential.	1. 633 2. continuous 3. 5/ 7,5/ 10 mW 4. 0,51 / 0,765 / 1,020 mW/cm^2^ 5. - 6. 5, 10 7. 0,156, 0.312/ 0.234, 0,468/ 0,312, 0,624, J/cm^2^ 8. - 9. - 10. homogenously over the entire dish	Siqueira A.F.P. *et al.,* 2016 [57]
16.	Sperm, ram and fish (tilapia)	UV or blue light generates high levels of ROS, resulting in a decrease in motility and fertility. In tilapia sperm, red and white light, which induce low levels of ROS, were found to improve motility and fertilization, while in ram sperm, only red light slightly improved the motility to a small extent.	1. 400-800 / 660 / 360 / 294 2. - 3. 40 / 10 / 1,5/ 0,1 mW/cm^2^ 4. - 5. - 6. - 7. - 8. 9. - 10. -	Zan-Bar T. *et al.,* 2005 [59]
17.	Sperm, boars	Structural changes in boar semen elements, mainly in the lipid component.	1. 633 2. - 3. 0,7-08, mW 4. - 5. - 6. 15, 30, 60, 120 ? 7. - 8. - 9. - 10. -	Lisichenko N.L. *et al.,* 2000 [61]
18.	Spermatozoa, eggs, fertilized eggs, embryos, and larvae of Sea Urchin *Paracentrotus lividus*	LILI does not induce morphological damage on the irradiated P. lividus gametes whose zygotes generate normal embryos and larvae. Overstimulation of some sperm leading to an accelerated cleavage of sea urchin zygotes is not deleterious to a correct embryogenesis.	1. 808 2. continuous 3. 1 /3 W 4. - 5. - 6. - 7. 64 /192 J/cm^2^ 8. - 9. - 10. in contact and perpendicular to the surfaces of an Eppendorf tube or a chamber of a multiwall plate	Amaroli A. *et al.,* 2017 [62]

1 wavelength, nm

2 mode of laser operation of the

3 power

4 power density

5 frequency, Hz

6 exposure per 1 zone (total time of procedure), min

7 energy density, J/cm^2^

8 number of procedures per course

9 periodicity

10 technique

* sequence of the presentation of laser illumination parameters

It is the increase in Ca^2+^ concentration, including that caused by laser illumination, that stimulates the work of the mitochondria and the synthesis of ATP [2, 63], which plays a key role in providing motility of spermatozoa [64-66]. The relationship between the Ca^2^+ -dependent release of NO (nitrogen oxide) in illuminated spermatozoa (optimal exposure 5 minutes) is also indicated with an increase in their activity [67], although it is more likely that this is only a secondary effect.

Most of the experiments were performed *in vitro,* but there are exceptions. In particular, M.D. Porras *et al.* (1986) [68] showed an increase in the number of spermatogonia and activation of spermatogenesis after the irradiation of mice testes with continuous infrared LILI. A significant increase in the production of testosterone by the interstitial cells of the testes of mice (Ley-dig cells) is also reported as a result of laser illumination by a red continuous LILI with a 633 nm wavelength [69-71].

Taha M.F. and Valorejerdi M.R. [72] performed laser illumination with continuous LILI with a 830 nm wavelength in modulated mode (power 30mW, frequency 300Hz) directly on the testes of Wistar rats. Both stimulating and spermatogenesis-inhibiting effects were demonstrated, depending on the power density and exposure of laser light. Errors of predecessors were repeated by other authors many years later, already working with completely unacceptable parameters on the testicles of rams and getting the expected negative result [2, 73]. Two important conclusions can be drawn from these studies: you do not need to concentrate the laser beam at a point, and it is also impermissible to shine for more than 1.5 minutes per zone. It is also not difficult to understand that exposure to high-intensity UV light is detrimental to cells [74]. Therefore, the selection of parameters for laser illumination in order to activate life processes must be approached with caution and preliminary justification.

Numerous studies indicate a direct relationship between an increase in intracellular Ca^2+^ concentration and the stimulation of the fertilizing capacity of spermatozoa in both animals and humans [48-51, 75-83]. It should be noted that in a number of works, conclusions are drawn (erroneous, in our opinion) about the leading role of reactive oxygen species (ROS) in the mechanisms of the biomodulating action of LILI [78, 79, 83-86]. But this is completely wrong, ROS are only secondary products of laser-activated cellular metabolism [2; 63], that is, a consequence, not a cause.

An increase in the concentration of Ca^2^+ causes the formation of ROS and the activation of antioxidant system as a whole, and not vice versa [87]. This is indirectly confirmed by the fact that the kinetics of the release of ROS depends on the energy, rather than on the power of LILI, and the most important component is the exposure [88], which can not be in the case of a direct photochemical reaction, when the power of a light source is more important. Moreover, direct experiments showed that ROS are released under the action of LILI by activation of Ca^2^+ -dependent mechanisms [89, 90].

Laser biomodulation appears to be more efficient and less expensive technology, which can be used with a fairly good scientific basis to improve artificial insemination and the effectiveness of embryonic systems [38]. As a result of laser illumination *in vitro,* the quality of the semen of bulls, rabbits and poultry used increases after a prolonged storage in the frozen state: spermatozoa penetrating ability (capacitation) is increased, their acrosomal reaction is induced with decreasing percentage of dead cells [46, 47, 91-95].

It is necessary to pay attention to studies in which it has been shown that laser illumination with continuous LILI of the red spectrum (633 nm, 10 mW, light spot area 0.125cm^2^, exposure 1-5 s) of immature oocytes of cows *in vitro* negatively affects the process of their maturation [96], although this was not observed in other analogous observations [97-102]. Perhaps the whole point is in the parameters of the illumination techniques and the differences in the experimental models. This question needs to be studied additionally, but it is necessary to understand that exposure to laser light with high energy density can harm or even kill the embryo. This is a fact that has been known for a long time [103]. Therefore, to ensure the safe operation of lasers, it is necessary to be guided by the relevant regulatory documents, the data of numerous studies and common sense.

Another important well-known fact is that the emergence and passage of dozens (up to 50) of waves of an increased concentration of calcium ions released from the endoplasmic reticulum depot is the essential condition for fertilization throughout the entire ovum volume [104]. The mechanisms of realization and the physiological necessity of this are still unknown, although the phenomenon has been actively studied for many years [105, 106], but one thing is clear that LILI realizes its biomodulating properties through activation of Ca^2+^ -dependent intracellular reactions, activating the same calcium depot. Consequently, laser illumination can potentially interfere with fertilization breaking the calcium transitions from the bound to the free state and vice versa. Perhaps, such specific processes peculiar only to oocytes somehow participate in the process of their maturation. While this is not known, we will therefore adhere to the point of view that we should avoid using any laser treatment technology on oocytes and egg cells.

Data from research conducted for animal breeding can be also used in medicine. Moreover, there is quite convincing evidence that low-intensity, both laser and incoherent light, can significantly improve the survival, motility and speed of movement of human spermatozoa [77, 79, 107-122].

## 4. Optimal selection of wavelength and laser mode

In most studies conducted on animals, illumination was carried out almost exclusively by continuous LILI in the red spectrum (633-650 nm), and much less frequently in other spectral ranges (Table 2).

**Table 2:** The wavelengths of light sources in experimental studies on the properties of spermatozoa.

Wavelength, nm	Sources
532	[39]
633-637	[43]; [47]; [57]; [78]; [91]; [94]; [96]; [107]; [123]
647	[56]
655-660	[40]; [41]; [92]
780	[50]
890-904	[111]; [112]; [114]

However, laser light with such parameters is impossible or almost impossible to use effectively in the clinic due to purely biophysical features (small depth of influence). Part of the problem is solved by using a different kind of light guide to deliver light energy to the right place through the cavities, for example, rectal illumination of the prostate gland, and the full use of LLLT is possible with pulsed LILI of the red and infrared (IR) spectrum [2, 124]. It is important that the general patterns obtained from experimental studies are reproduced qualitatively in the clinic.

Only one study used a pulsed infrared laser (905 nm wavelength) with a power of 50W (pulse duration of 200 ns), power density of 50W/cm^2^, and even with frequency of 10,000 Hz, motility increased and there was an absence of DNA damage. Probably, a positive result was obtained due to a small exposure time (30 seconds), and it was absent in normo-and asthenospermia, and was observed to be very significant (8.4 times), only with oligoasthenoteratozoospermia 30 minutes after laser illumination [114]. This confirms the well-known opinion that the degree of influence of LILI correlates with the severity of existing disorders (diabetes, neuropathy, *etc.)* [2]. There could not be a negative impact on DNA, even with such clearly overestimated energy parameters.

The absence of damage to the DNA of human spermatozoa has also been established for a continuous LILI of the red spectrum (wavelength 633 nm), even at a sufficiently high-power density (31mW/cm^2^) the illumination was carried out for 30 minutes (!). Even more so, the motility of spermatozoa has increased insignificantly [120]. At the same time, it is known that the effect of LILI can effectively protect the reproductive system from external stress factors (immobilization stress by single binding of rats for 6 hours in the position on the back) [125, 126], as well as from the pathogenic effect of radiation (such as disorders of spermatogenesis in the form of partial blocking of the formation of mature spermatozoa from postmeiotic cells (spermatids) and the development of destructive processes at the cellular and subcellular levels) [127-129].

Negative effects on the reproductive system of male mice (decrease in the concentration and the activity of sperm dehydrogenases), while maintaining fertility, are manifested only after illumination 5 times a week continuously for 4 hours a day with completely prohibitive and unsafe parameters: wavelength 1064 nm, pulsed mode, power 5MW, light pulse duration 12 ns, frequency 12.5 Hz, pulse energy 0.03J, and average power 360 mW only after 35 days [130]. In other words, in order to damage the organism using laser light using correct parameters, you need to try very, very hard.

S.V. Goryunov (1995, 1996) [111, 112] unequivocally showed that the optimal exposure, for both the wavelength of LILI 633 nm (continuous mode) and for 890 nm (pulsed mode, pulse duration100 ns, power 5 mW), the optimal exposure at which the sperm motility, their oxidative activity and cellular metabolism increases to the greatest extent is five minutes, while the pulsed mode is a bit more efficient [2], even with the fact that the laser light in the IR spectrum is absorbed less than in the red spectrum.

With regard to the choice of the optimal wavelength, opinions diverge. For example, it is shown that, when illuminated *in vitro,* sperm motility in men with asthenozoospermia rises on average by 4-5 times almost independently of the wavelength of the light source (470, 625, 660 and 850 nm) [113], and in studying the respiratory rate of spermatozoa of marine worms, a pronounced spectral dependence was observed in the wavelength range of 35-650 nm (maximum efficiency in the range of 400-430 nm) [44]. P. Gabel *et al.* (2009) [131] are convinced that the result is influenced by all exposure parameters: wavelength, power, exposure and coherence.

In the work of A.V. Stolyarov *et al.* (2002) [132], it was shown that artificial insemination of piglets with seed material after the preliminary illumination by LILI of different wavelengths (565, 595 and 660nm) allowed them to obtain the greatest increase in the number of pigs in the nest (+45%) at a wavelength of 595nm and exposure 0.5 min, and also good but less at 660 nm and one minute exposure (increase +25%).

We would also like to draw attention to the fact that all regularities were observed with direct exposure to spermatozoa *in vitro,* and when exposed to the patient's body, it is also necessary to take into account the anatomical features of the human body. Based on well-known generalized considerations, in particular, the understanding of biophysics of the processes of absorption and scattering of laser light, for clinical practice, the wavelength of 635 nm (red spectrum) is most often chosen when exposed to tissues and organs located at a depth of up to 5cm, and 890-904 nm (IR spectrum) when they are deeper (up to 15 cm) [2, 124].

The choice of spectral range data is also determined by the fact that it is in the regions of 600-650 nm and 850-900 nm, the absorption of light by spermatozoa is the most pronounced [111, 112].

## 5. Clinical studies

It should be noted that if the experimental studies on the influence of LILI of various *in vitro* and *in vivo* models which are somehow related to infertility are mostly by foreign authors, then clinical studies are performed almost exclusively by Russian scientists. Moreover, in Russia there is already very considerable practical experience of the use of laser therapy for these purposes.

In one of the few foreign clinical trials, the testes of men with oligozoospermia aged from 29 to 43 years old were illuminated with red continuous (633 nm, 12.5 mW) and pulsed infrared LILI (904 nm, a matrix of 5 laser diodes, a pulsed power of 12 W, frequency 800 Hz) for four minutes, twice a week, for only 10 sessions. Libido increased in 15 out of 20 patients alongside a significant improvement in the quality of sperm (increasing their motility and total number, reducing the number of abnormal ones) [133].

A.I. Gladkova (2011) [134] presented the results of her own multi-year experimental and clinical studies, as well as the work of her colleagues, which were used to substantiate the possibility of using laser therapy in andrology, showed the influence of various methods of laser influence on sexual behaviour, hormonal homeostasis, spermatogenesis and ability to fertilize [135-142].

Many researchers draw attention to the fact that the impact of pulsed infrared LILI with a transrectal delivery of laser light energy is preferable in the treatment of patients with chronic nonspecific prostatitis. Variation in frequency depending on the activity of the inflammatory process in the prostate gland allows to individualize therapy for patients with chronic obstructive pulmonary disease and to achieve better treatment results. LLLT in combination with traditional treatment is characterized by the more effective and rapid relief of the main symptoms of chronic obstructive pulmonary disease and a reduction in the frequency of complications [143-147]. The effect of traditional methods of treatment is intensified and potentiated through the generalization of the effect and the complex response of all homeostasis systems. The immuno-correcting action of LILI is caused by the stimulation of leukopoeza, including T-lymphocytes, which contributes to the rapid elimination of pathogens of urogenital infection. In this case, the number of patients with oligozoospermia after the treatment course decreases by more than 2-fold, and with asthenozoospermia by almost 4-fold [143-147]. In addition, laser action exerts a disaggregating effect on sperm, similar to the hypocoagulation effect of LILI on blood, which as a result improves the fertilizing properties of seminal fluid [19].

In another work, the laser therapeutic device with two infrared laser emitters (wavelength 890 nm, pulse power up to 10 W, 80 to 3000 Hz) was used. According to a method which based on the experience of other researchers using laser therapy, all patients underwent daily laser illumination on both testes simultaneously in the lateral and longitudinal projections for 10 days. This effect in the form of monotherapy with varicocele increases the concentration of active-mobile forms of spermatozoa from 25% to 37% and the number of morphologically normal forms from 27% to 39%. With idiopathic infertility, the use of local laser therapy causes an increase in sperm motility from 19% to 34% and an increase in the number of morphologically normal forms of spermatozoa from 13% to 23% [148-152].

This data is corroborated by the results of the studies, where the method was carried out in a similar manner, and the authors recommend that ligation of the spermatozoa by LILI prior to IVF is mandatory [153, 154]. Similar recommendations can be found in other works [105].

Clinical and experimental studies testify to the stimulating effect of LILI on the enhancement of the kinetic capabilities of spermatozoa and the functional-metabolic status of ejaculate neutrophils in patients with chlamydial infection, which can be used under appropriate clinical conditions [107-110].

For men of reproductive age who are in a partnership for more than one year, as well as those with symptoms of prostatitis, vesiculitis or epididymorchitis, it is necessary to conduct a clinical and microbiological examination to eliminate any hidden urogenital infections (chlamydia, trichomonias, mycoplasma genitalia, ureaplasma, herpes simplex virus, *etc.)* or sexually transmitted diseases, before the start of treatment, including an examination of all sexual partners. Laser therapy of patients with prostatitis and vesiculitis can eliminate infiltrative-exudative changes in the prostate gland, and the appointment depends on the stage of the inflammatory process in the prostate gland. Carrying out LLLT can improve the outflow of inflamed secretions from the glands of the prostate, increase local immunity, eliminate pain and dysuric symptoms and improve reproductive and copulatory functions [155, 156].

Since a direct link between the presence of epididymoortitis and infertility and the effectiveness of laser therapy in treating this category of patients is questionable [157-160], the inclusion of its complex recovery of male fertility is desirable.

A reliable large therapeutic effect that has positive, longlasting lasting results in the treatment of infertility in men with chronic inflammatory diseases of the organs of reproduction is the use of IR LILI. The local effect on the fields of projection of the sexual glands is 91.7%, and the use of laser acupuncture is 85.2%, compared with traditional medicinal therapy, which is 76.8%. The local effect allows increasing the number of actively mobile sperm forms in the ejaculation by 45-50%, removing the inflammatory process and restoring microcirculation in the sex glands. Exposure to acupuncture points (AP) (Pat. 2185211 RU) [161] of the lumbar region further increases the concentration and reduces the number of pathological forms of sperm in the ejaculate by 10-15%, improving the endocrine regulation of spermatogenesis. At the same time, a sufficient therapeutic effect is achieved after 5 procedures. An additional course of LLLT is carried out 6-9 months after the main course [162-165].

A.B. Ikhayev (2013) [166] applied a combined-correlated method of laser therapy in patients with chronic nonspecific prostatitis with infertility in a strong and strongly-medium sexual constitution. A vibro-magneto-laser massage of the prostate gland [31] with the rectal attachment VMLG10 (LILI+magnetic field+vibration) to the laser therapeutic device "Matrix-Urolog" (produced by Research Center Matrix, Russia) (wavelength 635 nm) every second day, five-minute exposure, laser illumination modulation frequency 10Hz, a course of 15 procedures. Patients with oligoasthenoteratozoospermia I-II stage with the duration of chronic abacterial prostatitis (CAP) up to 5 years and the age of up to 40 years, combined illumination therapy is also prescribed according to the method of local laser negative pressure (LLNP) [167; 168] for 12 minutes, every other day. This technique implies that a special flask with a laser head in the form of a ring is put on the penis. A vacuum (negative pressure) of 15-30 kPa is created and simultaneously lasers are illuminating. It repeats in cycles of 1.5 minutes. Under the influence of combined correlated illumination therapy, the algic syndrome is stopped in 75%, dysuric syndrome in 61%, erectile dysfunction in 54% and asthenic-neurotic in 59.4% of patients. Normalized prostate gland size occurs in 80% and pituitary-adrenal-testicular system in 65% of patients with chronic prostatitis with infertility. The experience of clinical application of the proposed method of LLLT for 12 months. showed that after the end of the treatment course, 67.5% of couples were pregnant [166].

High efficiency is also shown in intravenous laser blood illumination (ILBI) in the treatment of patients with CAP with impaired fertility. The Matrix-ILBI device (produced by Research Center Matrix, Russia), a wavelength of 635nm, power of 1.5-mW at the output of KIVL-01 (intravenous light guides produced by Research Center Matrix, Russia), for a course of 10 sessions of 10 minutes. 15 patients (37.5%) were in a strong sexual constitution, 14 (35%) were in the middle and 11 (27.5%) were in a weak sexual constitution, a prostate massage was also performed daily (for a course of 15 procedures). As a result of the treatment, normospermia was detected in 72.5% patients with strong and medium sexual constitutions. As a result of the treatment, the concentration of follicle-stimulating hormone (FSH) in the blood decreased by 28%, luteinizing hormone by 17%, estradiol by 17%, prolactin (PRL) by 38%, dehydroepiandrosterone sulfate - by 18%, testosterone - increased by 33.5%, taking the regulatory data (p > 0.05). As a result of treatment, the functional activity of the hypothalamic-pituitary-adrenal-testicular system occurred in 27 (67.5%) patients with a duration of CAP no more than 5 years. Within one year after the course of treatment, pregnancy occurred in 25 (62.5%) partnered couples in which men were between the ages of 22 and 40 with a strong and medium sexual constitution, with a duration of CAP ≤ 5 years [169-171].

With the main treatment regimens, it is recommended to perform laser acupuncture, the effect of LILI on AP of the lumbar region and balneotherapy (iodine-bromine baths) to improve efficacy [168, 172-175].

Based on these studies in Roszdravnadzor, a complex method of the correction of infertility in patients with chronic prostatitis was recorded [176].

Patients with reproductive dysfunction alongside even abacterial prostatitis are advised to use ultraviolet blood illumination (UVBI), which is more often used for various disorders of the immune system [29, 177, 178]. Currently, the LUVBI® (laser ultraviolet blood illumination) technique is done intravenously, using only LILI with a wavelength in the range of 365-405 nm and almost always combining every other day with ILBI-635 (wavelength 635 nm, power is 1-2 mW) [179].

If the hormonal function and spermatogenesis are impaired in men with obesity of no more than 2 grade, it is advisable to prescribe a combined treatment that includes the action of pulsed infrared LILI (890-904 nm) on the collar area (projection of the vertebral arteries at the C3-C7 level and the subscapular region according to the labile technique, scanning with a speed of 1cm/ s) and other physiotherapy methods alongside a standard complex (low-calorie diet, moderate exercise and long-term pharmacotherapy). In case of the violation of the copulatory function, it is advisable to prescribe to patients also a local effect on the testicles (in the lateral and longitudinal projections, 5 minutes for each testicle) and rectal fillings of pantocrine [180-182].

Regarding Slonimskiy B.Yu.[181], a complex treatment program in patients with obesity and impaired fertility can eliminate lipid imbalance, normalize some metabolic parameters, including the content of leptin and TNF-α, which is important for the restoration of fertility. There is a correction of erectile and copulative disorders in the form of recovery to normal values of neurohumoral, psycho-emotional, erectile and ejaculatory components, as well as indicators of erectile function, as is shown by an increase to the physiological norm of the cumulative index of the IIEF scale (The international index of erectile function) (from 14.3 ± 0.3 to 23.8 ± 1.2), improvement of the functional state of the central and peripheral hormonal structures, which is confirmed by the restoration to the values of the physiological norm of sex steroid hormones. After the therapeutic course, restoration of the spermatogenesis disturbed in the initial state is observed, manifested in an increase in the volume of the ejaculate, in the concentration of spermatozoa, in improving their shape and motility. A comprehensive reproductive function restoration program for obese men is a highly effective method, and the achieved therapeutic results in 78.8% of patients persist up to one year [181].

Analysis of the hormonal profile revealed a tendency to decrease FSH levels in patients with severe oligoastenoteratozoo-spermia from 11.5 mU/ml to 8.0 mU/ml, which indirectly indicated the influence of LILI on Sertoli cells [149, 183].

A special section of publications are patents, where the novelty of the method and/or the device is simultaneously protected, and the results of a study of their effectiveness are given. Patent search made it possible to identify nine patents, to some extent related to fertility, in which LILI is used. Since the full text of all publications is publicly available, only the main provisions are given in table form (Table 3).

**Table 3:** Patents in which LILI illumination is associated with various aspects of infertility.

**Purpose, goal**	**Laser exposure parameters**	**Impact localization, method**	**Patent**
Improving the quality of sperm production in male boars	Not specified	3 AP (acupuncture points) with loca-lization description	[183]
Treatment of men with the pathology of spermatogenesis	633 nm, continuous mode, 3-4mW	Corporeal AP: T3, T4, V23 + one of the auricular: AP22, AP23, AP32	[184]
Treatment of men with autoimmune infertility	365-400 nm, 20 mW (incoherent light), 30 minutes, 6 daily procedures	UVBI intravenously	[161]
Improvement of sperm quality in pathospermia in the IVF program	Pulsed infrared IR LILI, 890 nm, 3.5 W, 300-600 Hz, 7-10 minutes, 5-7 daily procedures	On the perineum and suprapubic region	[186]
Treatment of men with autoimmune infertility	660 nm, light-emitting diodes, modulated mode, frequency 1-5 Hz, 1 mW/cm^2^, 15-20 minutes with pauses, 10 daily procedures	The penis, LNP (local negative pressure + incoherent illumination)	[187]
Stimulation of spermatogenesis	635 nm, continuous operation, 30 mW, 10-15 daily procedures	Contact on the area of the scrotum	[188]
Treatment of men with impaired spermatogenesis	Not specified	UVBI	[189]
Stimulation of spermatogenesis in the experiment, non-native male rats Increase in the functional and metabolic status of human spermatozoa	475 nm, continuous mode, 10 mW/cm^2^, 1 minute, 10 daily sessions 635 nm, modulated mode, frequency 100 Hz, 10 mW/cm^2^, 1 minute	On the testicles Spermatozoa derived from healthy human semen *in vitro*	[190] [191]

## 6. Conclusion

Despite the active debates and discussions on the topic of the presence/absence of "full-fledged" diagnostics, the case of idiopathic sperm quality disorders in more than half of the cases of male infertility is unquestionable. Consequently, in the first place clinicians should consider the non-specific treatment methods aimed at "general improvement" that trigger the mechanisms of sanogenesis, restoration of disturbed homeostasis and normal physiological regulation.

Previously, it was thought that laser therapy was only of an auxiliary nature and is prescribed in conjunction with drug therapy or at the final stage of traditional treatment [192], but further studies completely refute this view. Analysis of the scientific literature suggests that laser therapy should be used as much as possible in the complex treatment of men with infertility, since the effectiveness of the method is not just high, but often has no alternatives. For laser illumination, it is best to use exclusively pulsed LILI, red (635 nm) and infrared (904 nm) for local illumination, alternating with continuous LILI with a wavelength of 635 nm (red spectrum) and 365 nm (ultraviolet) for intravenous laser blood illumination.

It is necessary to use the available low level laser therapy methods as widely as possible: local, rectal, laser acupuncture, ILBI, on the projection of various organs, paravertebrally, *etc.,* while setting all parameters of the laser (wavelength, mode of operation, frequency for pulsed lasers, power, density power determined by the method of exposure, exposure, localization), which are established by appropriate regulatory documents and clinical recommendations [3, 193].
